# MOGADOC: a database with 3D-structures

**DOI:** 10.1186/1758-2946-6-S1-P3

**Published:** 2014-03-11

**Authors:** Jürgen Vogt, Evgeny Popov, Rainer Rudert, Natalja Vogt

**Affiliations:** 1 Chemical Information Systems, University of Ulm, 89069 Ulm, Germany

## 

The MOGADOC database (Molecular Gas-Phase Documentation) has grown up to 11,500 inorganic, organic, and organometallic compounds, which were studied in the gas-phase mainly by microwave spectroscopy, radio astro-nomy, and electron diffraction. The database contains about 9,000 numerical datasets with internuclear distances, bond angles and dihedral angles. Most of the corresponding molecular structures are also given as 3D presentations (ball-stick-models). The retrieval features of the HTML-based database have been described elsewhere in the literature [1,2]. Some years ago a Java-applet has been developed, which enables the 3D-visualization of the molecular structures. The user can interactively rotate, shift and scale the 3D-models and can “measure” bond lengths as well bond, dihedral and elevation angles [3].

**Figure 1:**
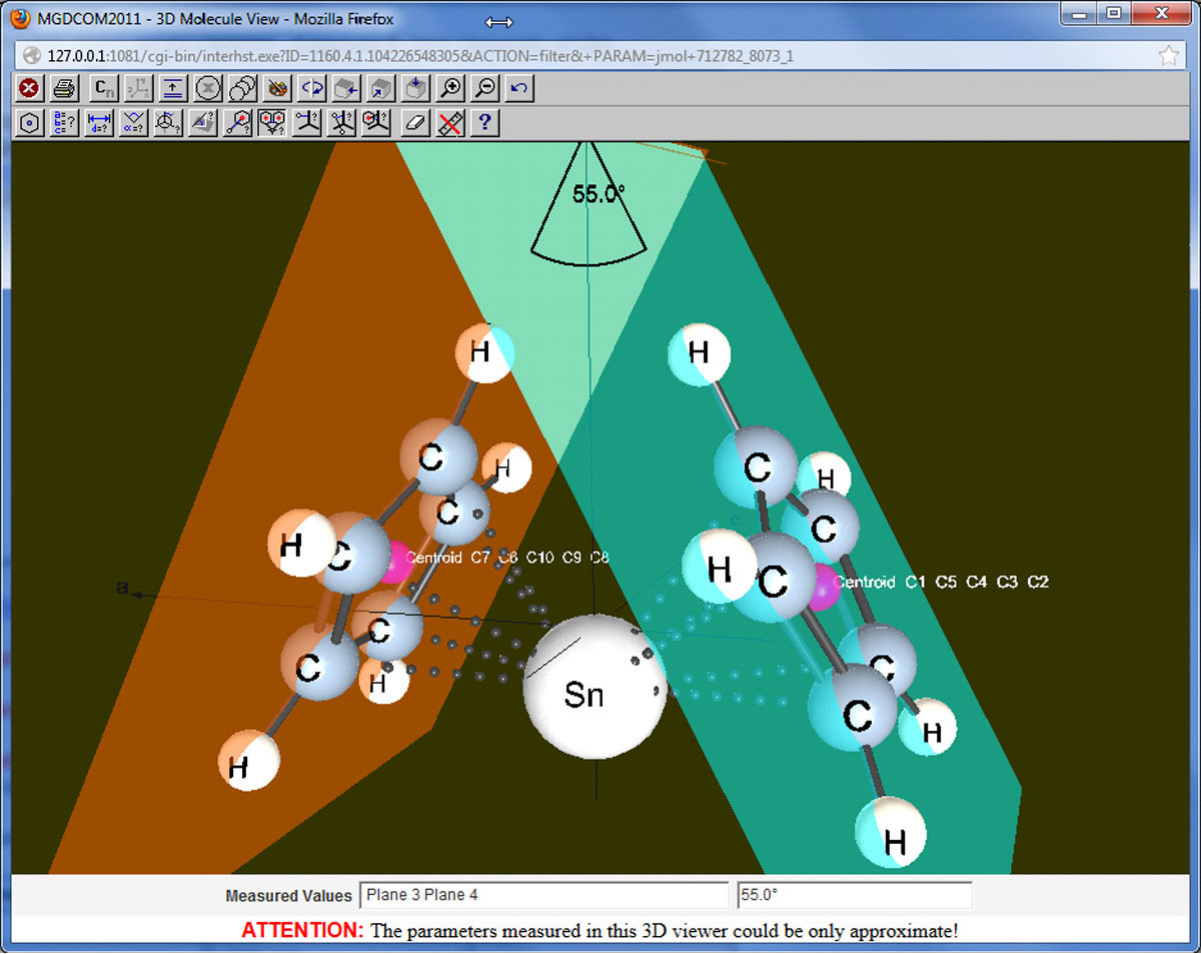


Recently new “measurement” features have been supplemented (such as for distances between centroids, angles between ring planes, etc.).

The project has been supported by the Dr. Barbara Mez-Starck Foundation.
